# Evaluation and Association of Hematological and Biochemical Parameters of Untreated COVID-19 Patients on the basis of Differences in Ethnicity and Curcumin as a Possible Treatment

**DOI:** 10.2174/1871530323666230314121123

**Published:** 2025-07-10

**Authors:** Munir Akhtar, Rubaida Mehmood, Mukhtar Hussain, Momna Warraich, Hafeez Ullah, Zahida Batool, Sumara Ashraf, Rida Sana

**Affiliations:** 1 Biophotonics Imaging Techniques Laboratory, Institute of Physics, The Islamia University of Bahawalpur, Bahawalpur, 63100, Pakistan;; 2 Head Labs Sections, MINAR Cancer Hospital, PAEC, Multan, Pakistan;; 3 Quaid-e-Azam Medical College, Bahawalpur, Pakistan;; 4 Shifa International College of Medicine, Islamabad, Pakistan;; 5 Department of Physics, Women University of Multan, Multan, Pakistan;; 6 Department of Chemistry, NFC Institute of Engineering and Technology, Multan, Pakistan

**Keywords:** Curcumin, COVID-19, serum electrolytes, HGB, urea, creatinine

## Abstract

**Background:**

CBC (complete blood count) tests, along with RPM (Renal parameters) and LFT (Liver function tests), are clinically important for coronavirus patients; curcumin can serve as a possible treatment for SARS- CoV.

**Objective:**

The objective of the study was to determine the relationship of CBC parameters with renal parameters and liver function tests and to develop the hypothesis that curcumin may be the best and non-invasive drug for coronavirus.

**Materials & Methods:**

The differences between the results of 91 confirmed cases of COVID-19 (symptomatic and asymptomatic) and 100 controls were assessed by an independent t-test and Mann-Witney U Wilcoxon test. Microscopy, hematological tools, and techniques were used to assess the improvements/abnormalities in blood components and parameters.

**Results:**

This is a case control study along with the feasibility of curcumin as COVID treatment. The association between parameters was assessed by Pearson & Spearman correlation analysis. The level of significance was *p* < 0.05. Changes were observed in urea (*p* = 0.000), creatinine (*p* = 0.02), total bilirubin (*p* = 0.000), SGPT (ALT) (*p* = 0.000), RBC (*p* = 0.001), HGB (*p* = 0.001), MCV (*p* = 0.002), MCH (*p* = 0.03), MPV, PDW, NE%, LY%, and MO% EO% (*p* = 0.00), in comparison to normal controls. Differences in the correlation of electrolytes, RPM, and LFT tests along with CBC parameters in Pakistani and Chinese individuals provided a new idea for using various diagnostic and therapeutic tools in different ethnic groups. The COVID-19 infected blood components and parameters showed rapid improvement/recovery, especially the total count of platelets and WBCs (lymphocytes and basophils), HGB, HCT, MCV, and MCH.

**Conclusion:**

Curcumin drugs can be used as an immediate remedy/treatment to cure COVID-19 patients.

## 1 INTRODUCTION

COVID-19 is the current pandemic that has a devastating worldwide impact. It originated in Wuhan, China, in December 2019 [1] and has caused 932,715 global deaths with 29,440,414 total reported cases to date (September 15^th^, 2020) [2]. In Pakistan, the first case of COVID-19 was officially confirmed on 26^th^ February, 2020 by the government [3]. In the coming months, it has spread to almost all over the country with 302,020 total reported cases, 289,806 recoveries, and 6383 mortalities [2]. Laboratory tests such as complete blood counts (CBCs) play a vital role in the diagnosis and pathophysiology of infectious and respiratory tract diseases [4]. Complete blood count (CBC), one of the most routinely used blood tests, is used to assess the production and concentration of different types of blood cells. These include RBCs and various subgroups of WBCs like neutrophils, lymphocytes, *etc*. It provides quantitative data on RBC indices, hemoglobin, and hematocrit. Immune function can also be checked by CBC as it provides data of WBC counts with differentials. The quantitative information is provided in CBC report, in which all indices and values of blood parameters are written with their normal reference values. CBC is a commonly conducted lab test in hospitals, but the data it provides is seldom used to its full potential to provide significant conclusions about disease prognosis. Instead, it is used by clinicians mainly to assess basic information like the diagnosis of anemia and infection or confirm normal homeostasis in the patient [5]. However, a CBC test can provide much more significant data for disease pathophysiology if it is diagnosed properly and correlated with other patient parameters, observations, diagnostics, and signs and symptoms. CBC consists of many useful blood parameters like; WBC and RBC counts, hemoglobin level, hematocrit, mean corpuscular volume (MCV), mean corpuscular hemoglobin (MCH), mean corpuscular hemoglobin concentration (MCHC), red blood cell distribution width (RDW), platelet blood count (PLT), platelet crit (PCT), mean platelet volume (MPV), platelet distribution width (PDW), neutrophil extracellular traps (NETS), lymphocytes, eosinophil, monocytes, and basophils. Their quantitative data can be utilized to make the diagnosis and predict the course and outcomes of many diseases [6]. The most important parameter in the CBC blood test is the hemoglobin level. It is responsible for the transportation of oxygen to different tissues and organs of the body (normal concentration in females =12.0 - 15.5 gm/deciliter, normal concentration in males = 13.5 - 17.5 gm/deciliter). Its clinical significance is also emphasized by its deficiency causing anemia that may be caused by bleeding or iron deficiency disorders, leukemia, vitamin-related, chronic diseases, folate deficiency, kidney and liver disease, hypothyroidism thalassemia, *etc*. Like hemoglobin, lymphocytes (B & T cells) have a major impact on CBC. The normal lymphocyte value in children and adults is 3,000 - 9,500 and 1,000 - 4,800 in 1 µl of blood, respectively. It helps in the development of the immune system of the body and protects against infections. Lymphocytes activate as the first sign of infection, trigger immune responses, and help in the development of immunity against past infections and vaccinations [7]. T-cell deficiency may result in infections from fungi, viruses, and parasites, while B-cell deficiencies may lead to several infections. Thus, this count is a disease predictor in COVID-19 patients [7]. To find the treatment for this infection, many marketed drugs were prepared after many elucidations performed on this virus’s genome. Protease is the main selection of drugs used for the treatment of COVID-19. Protease acts during the replication of RNA of the coronavirus [8]. One of the most promising antiviral potentials is curcumin. Curcumin is a polyphenol having three types, i.e. curcuminoid (curcumin I) 77% demetoxicurcumin (curcumin II) 17% and bisdemetoxicurcumin (curcumin III) 3% which is obtained from rhizome known as Curcuma longa [9]. Curcuma longa is the source of Curcumin. Curcumin has many biological targets like antidepressants, antiviral, anticancer, antioxidants, and anti-inflammatory [10-13]. During COVID- 19, immunological inhibition and inflammatory response of Curcumin can reverse edema and pathways of the pulmonary system assisted by fibrosis [14, 15]. Curcumin has its effects on many organs, cells and diseases like T-Cells, liver, neurological diseases, inflammatory diseases, kidney diseases, lungs diseases and other diseases. Curcumin can boost the anticancer function of effector T-cells in addition to inhibiting the exhaustion of T-cells during COVID-19, T-cell improvement, and proliferation in tumours [16]. The metabolic syndrome and liver diseases caused by high intake of fructose and curcumin can improve these defects by using radiolabelling techniques [17]. Colitis is an inflammatory disease during which lymphocytes, neutrophils, chemokinesis, cytokinesis and macrophages are destroyed by NLRP3 activation. Curcumin deactivates the NLRP3 by release of cathepsin B, K(+) efflux and production of intercellular ROS [18, 19]. A neurological abnormality is epilepsy caused by inflammation. A study on rats show that activation of NLRP3 and IL-1β are the causes of immunopatogenesis of disease and curcumin deactivates NLRP3 and reduces the severity of this disease by inhibiting inflammation [20, 21]. Nephritis and hyperuricemia in rates can be removed by using curcumin. Curcumin inhibit the NLRP3 and lowers the level of cytokinesis in Nephritis and hyperuricemia abnormalities of kidney [22]. Inflammation and oxidative stress causes acute lungs injury (ALI). Curcumin reduces ALI by inhibiting NLRP3 and lowers the level of cytokinesis [23]. We have tried to study blood components and blood parameters of COVID-19 patient blood with five different concentrations of curcumin ranging from 0 mm to 20 mm with a step size of 5 mm hematologically.

## 2 MATERIALS AND METHODS

In this research, CBC, blood smear, platelet-rich plasma, and microscopy are the hematological techniques used to discuss the abnormalities or improvement in cell count, shape, and size under 0 mM, 5 mM, 10 mM, 15 mM, and 20 mM of curcumin.

### 2.1 Sample Set for Blood Smear

Five different samples, each of 3 ml of confirmed COVID-19 female patients, were taken in an anticoagulant tube (EDTA). Then five varying concentrations, *i.e*. (0 mM, 5 mM, 10 mM, 15 Mm, and 20 mM) of Curcumin were admixed separately in each tube, and CBC was performed. Here 0 mM refers to the inherent value of Curcumin *i.e*., to no curcumin. Slides of each sample were made by using ethanol for fixing and field stain (A, B) for staining. Then each slide/ smear was diagnosed/analyzed under white field microscopy by using a lens of 100 X at room temperature. An image of each smear was captured for viewing abnormalities or improvement in the shape and size of WBCs, platelets, and RBCs.

### 2.2 Laboratory Measurements

Patients visited Covid center of Bahawalpur Victoria Hospital, Multan, for diagnostic and therapeutic purposes and completed a structured form concerning demographic characteristics. Samples were taken in K2-EDTA vials (Bd Vacutainer for TFT’S & CBC analysis) [24, 25] and red top BD vials after the approval of a local ethical committee of MINAR Cancer Hospital (Pakistan Atomic Energy Commission, Ref. No. M-3(13)/ 2018) and informed consent of participants. Urea, Creatinine, total bilirubin, SGPT (ALT), and alkaline phosphatase were performed on the clinical chemistry analyzer. Selectra Junior by vital scientific B.V. Article No. 6002-900-410, Software version: 4.1.x Netherlands. Serum electrolytes (Na^+^, K^+^) were measured by using ion-selective electrode technology on an Easylyte analyzer (Medica Corporation, USA, 34440 CNKC). Routine hematology testing was performed on the MEK9100 Celltac G Hematology Analyzer [26].

## 3 RESULTS

Changes were observed in Urea (*p*=0.000), Creatinine (*p*=0.02), total bilirubin (*P*=0.000), SGPT (ALT) (*p*=0.000), RBC (*p*=0.001), HGB (*p*=0.001), MCV (*p*=0.002), MCH (*p*=0.03), MPV, PDW, NE%, LY%, MO% EO% (*p*=0.00) when compared with normal controls. Overall results showed a strongly significant negative association of HGB with urea (r-0.43, *p*=0.04), ALP with PDW (r=-0.9, *p*=0.03), Na^+^ with WBC (r= -0.6, *p*=0.04), K^+^ with MO% (r-0.75, *p*=0.007), EO% (r-0.83, *p*=0.004) and neutrophils (r=0.64, *p*=0.03). In Pakistani population, SGPT levels associated with NE% (r=0.82, *p*=0.007) and LY% (r=-0.83, *p*=0.006), Sodium level associated with EO% (r=-0.92, *p*=0.03). In the Chinese population, a strong association of serum electrolytes (Na^+^& K^+^) was observed with CBC parameters. Sodium (Na^+^) was associated with WBC (r=0.96, *p*=0.009), RBC (r=-0.95, *p*=0.01), HGB (r=-0.96, *p*=0.007), PLT (r=0.89, *p*=0.04) and NE% (r=0.92, *p*=0.02), Potassium was associated with WBC (r=0.97, *p*=0.006), HGB (r=-0.94, *p*=0.02) and LY% (r=-0.90, *p*=0.03). SGPT was associated with HGB(r=0.64, *p*=0.007), Alkaline phosphatase with MO%(r=0.51, *p*=0.04) and EO% (r=-0.62, *p*=0.01)), urea and creatinine both had a negative association with WBC (r=-0.53, *p*=0.03), (r=-0.51, *p*=0.04) respectively, and both were associated positively with HGB (r=0.73, *p*=0.001), (r=0.81, *p*=0.00), HGB was also strongly associated with total bilirubin (r=0.72, *p*=0.002) as shown in Tables **1** and **2**. A strongly significant positive association of RBCs with creatinine (r=0.86, *p*=0.03) was observed. The study was performed on 91 confirmed cases of COVID-19 (symptomatic and asymptomatic) with ages ranging from 11-63 years, mean age ± SD (34.89 ± 19.19), 16 were females and 75 were males, and 100 healthy controls were taken for comparison. Then their hematological parameters (WBC, RBC, HGB, HCT, MCV, MCH, MCHC, PLT, RDW, PCT, MPV, and PDW) and biochemical parameters as RPM, LFTs.Serum electrolytes (Na^+^ and K^+^) were also monitored. The comprehensive results are shown in Table **1**. Significant differences recorded by the Mann-Witney U Wilcoxon test for Urea (*p*=0.000), Creatinine (*p*=0.02), total bilirubin (*p*=0.000), SGPT (ALT) (*p*=0.000), RBC (*p*=0.001), HGB (*p*=0.001), MCV (*p*=0.002), MCH (*p*=0.03), MPV, PDW, NE%, LY%, MO%, EO% (*p*=0.00) when compared with normal controls as shown in Table **1**.

### 3.1 Statistical Analysis

For statistical analysis, SPSS version 24.0 (SPSS Inc., Chicago, IL, USA) was used. The significance of the differences between COVID-19 patients and the healthy control group were assessed by independent t-test and Mann-Whitney U Wilcoxon test after checking for normality by Kolmogorov-Smirnov and Shapiro-Wilk tests. Association between parameters was assessed by Pearson & Spearman correlation analysis. The level of significance was *p* < 0.05.

## 4 DISCUSSION

### 4.1 Laboratory Parameters

In this study, we have focused on the hematological and biochemical parameters of COVID 19 patients related to two different ethnic groups, *i.e*., Pakistani and Chinese. We have analyzed and summarized the lab tests of COVID-19 patients, especially biochemical and hematological parameters, and serum electrolytes of people related to both populations. Changes in urea and creatinine levels of COVID-19 patients in both populations were observed when compared with control groups, and these changes may result in higher mortality rates among patients with COVID 19 in both populations [27]. Changes were also observed in liver enzymes, especially ALT and total bilirubin, but not in ALP, indicating the injury to hepatocytes caused by COVID 19. Ikeagwulonu, Richard Chinaza, *et al*. proved an increase in both ALT and total bilirubin, but we have observed an increase in ALT levels and a decrease in total bilirubin level [28], which indicates the changes observed in LFT by the viral attack of both populations. Among CBC parameters TLC remained unchanged, but strongly significant changes were observed in Differential leukocyte counts (NE%, LY%, MO%, and EO%), a previous study proved marked neutrophilia and lymphopenia in hospitalized COVID-19 patients [29]. Eosinopenia was observed at the onset of COVID 19, but afterward, the average value of eosinophils continued to increase significantly, as proved by Tian *et al*, we also observed an increase in Eosinophil counts [30] MO%. Among the hematological markers, significant changes in RBC count, hemoglobin level, MCV, and MCH were observed. All parameters were decreased significantly, but a study has proved that the decrease in MCV and MCH levels shows the severity of the disease, in another way, they also indicate normal levels of RBC counts and HB concentration in COVD-19 patients. This was not evident when we studied two different populations [31]. Changes were also observed in MPV and PDW, but platelet count remained within the reference ranges.

### 4.2 COVID-19 and Curcumin

Detailed and depth study about abnormalities/improvement in shape and size of blood cells and parameters for five samples having concentrations,*i.e*., (0 mM, 5 mM, 10 mM, 15 mm, and 20 mM) of curcumin with the help of images of slides and CBC. WBCs count/µL for the inherent value of curcumin is 8.5 ×10^3^/µL and at optimum concentration, WBCs count is 8.94 × 10^3^/µL and at same optimum concentration *i.e*. 20 mM after 30 minutes WBCs count is 12.25×10^3^/µL as shown in Table **3**. Thus, there is an improvement in the count of WBCs, as shown in Fig. (**1a**, **b**). Out of the five types of WBCs, lymphocytes, and basophils show demonstrative changes. Lymphocytes are natural germ killers and remove the damaged/destroyed parts affected by the virus or cancer and can produce antibodies. Curcumin improves the count of lymphocytes from 2.10% to 4.69%. Basophil produces heparin (an anticoagulant) and histamine (a homeostasis factor). Basophil cell count was also increased with increasing concentration of curcumin from 0.45% to 2.20%. Other types of WBCs do not show noticeable changes. The size and shape of WBCs were irregular, as shown in Fig. (**2a**) and they became regular in shape and size with increasing concentrations of curcumin as shown in Fig. (**2b**-**e**). RBC count is higher in COVID-19 patients but curcumin lowers the RBC count with increasing concentration of curcumin to the optimum level as shown in Fig. (**1**). The shape of RBCs changes from biconcave to spherical and spiked with increasing concentrations of curcumin, as shown in Fig. (**2c**-**e**). Platelet cell count was also increased in covid-19 patients by admixing curcumin. Blood without curcumin has 178.2×10^3^/µL of platelet cells and reaches 249.7×10^3^/µL at 20 mM concentration. Transportation of oxygen in different parts of the body, and the red color of blood is detected by a pigment/parameter known as HGB. HGB was 10.84 g/dL at the inherent value of curcumin and increased up to13.94 g/dL at the optimum value as shown in Table **3** and Fig. (**1b**). Parameters like HCT, RDW-CV, PCT, PDW, and P-LCR (%) increased to around 4-6%. Some parameters like MCV, RDW-SD, and MPV (fL) increase from 0.4 to 4 fL. MCH was 26.9 at the inherent value of Curcumin and reached 30.5 pg at the optimum value of curcumin. MCHC was 29.3 g/dl at the inherent value of Curcumin and reached 32.1 pg at the optimum concentration of curcumin as shown in Table **3**.

### 4.3 COVID-19 and RPM Parameters

Renal abnormalities occurred in the majority of patients with COVID-19 pneumonia. Although proteinuria, hematuria, and AKI often resolved in such patients within 3 weeks after the onset of symptoms, renal complications in COVID-19 were associated with higher mortality [27].

In conclusion, the thromboembolic consequences of COVID-19 can occasionally result in rare complications such as aortic thrombosis and renal infarction. During pandemic, we advise that physicians should maintain a high degree of clinical suspicion to diagnose rare manifestations of this novel disease for timely management [32].

In summary, we provided evidence that kidney impairment is common among COVID-19 patients, and its clinical manifestation is abnormal urinalysis, indicating that a urine routine test is a better indicator to unveil potential kidney impairment than a blood chemistry test. Furthermore, our results suggested that urinalysis is a useful tool to predict disease severity. Thus, we call for front-line healthcare workers to pay more attention to kidney impairment in patients with SARS-CoV-2 infection and routinely monitor urinalysis to judge potential kidney impairment and evaluate disease severity [33].

Among a cohort of 42 patients dying from COVID-19, the histologic evaluation revealed the acute tubular injury, which was typically mild relative to the degree of creatinine elevation. These findings suggest the potential for reversibility upon resolution of SARS-CoV-2 infection [34].

In our study, we found that the dynamic changes in these kidney function indicators were associated with different severity and outcomes of COVID-19. Importantly, the sharp differences in the dynamic changes in the three markers of kidney function in nonsurvivors *versus* the relatively stable patterns observed in the survivors implied a significant association between the aggravation of kidney injury and the deterioration of the disease and death in the pathogenesis of COVID-19. Similar results were observed in the male and female subgroups, with more significant trends in males than in females. It is known that estrogen affects ACE2 expression in the kidney, which could be one of the reasons for gender-related differences in the dynamic patterns of kidney parameters during hospitalization observed in our study [35].

The incidence of abnormal urine analysis and kidney dysfunction in COVID-19 was high, and AKI is closely associated with the severity and prognosis of COVID-19 patients. Therefore, it is important to increase awareness of kidney dysfunction in COVID-19 patients [36].

Our findings show the prevalence of AKI on admission in patients with COVID-19 is high and is associated with a greater in-hospital mortality rate. Physicians should closely monitor any patient with impaired renal function on admission, regardless of respiratory status. Our data comes from a multicenter registry and therefore does not allow having a precise mortality risk assessment. More studies are needed to confirm these findings [37].

We have found that over one in five patients developed AKI-RRT, over 60% of whom died within 28 days. Among those with AKI-RRT who survived hospital discharge, nearly one in three remained RRT dependent. We identified several patients and hospital-level risk factors for AKI-RRT and death. Future studies are needed to investigate the long-term outcomes of patients with AKI-RRT in the setting of COVID-19, as well as to explore the underlying etiologies of AKI in this setting so that targeted therapies can be developed [38].

Our study found a high prevalence of renal impairment in patients with COVID-19 infection. The results also demonstrated a significant correlation between renal function indicators and derangement in different laboratory markers, including hematological indices and liver enzymes. In addition, the severity of renal impairment was significantly associated with a more severe clinical course and subsequently increased the risk of ICU admission. This highlights the importance of evaluating renal function and renal function indicators as predictive markers for COVID-19 progression. Hence, the study findings might help in early and effective intervention for high-risk COVID-19 patients, improving the disease outcomes and subsequently reducing COVID-19-associated morbidity and mortality [39].

The dynamic changes of the three kidney function markers were associated with different severity and poor prognosis of COVID-19 patients. BUN showed a close association with and a high potential for predicting adverse outcomes in COVID-19 patients by severity, stratification, and triage [35].

### 4.4 COVID-19 and LFTS

Liver injury is associated with COVID-19 infection, especially in its severe form, and earlier detection and reversal of these effects can positively affect the patient’s prognosis and survival. A previous study that analyzed a cohort of patients who died of severe COVID-19 reported elevated levels of AST (79%), ALT (14%), and GGT (36%) in these patients [28].

Despite the common descriptions of liver enzyme abnormalities observed in COVID-19 patients, the frequency, intensity, and impact of liver injury are discrete and of little clinical significance regarding the morbidity or mortality of this disease. A better understanding of the natural history of liver involvement may be addressed soon with well-designed prospective studies regarding viral and immunologic research [40].

In conclusion, an abnormal liver profile is present in COVID-19 patients. SARS-CoV-2 may cause liver damage. Abnormal liver function is associated with elevated levels of inflammatory markers. Elderly and male patients with abnormal liver function were at higher risk of developing severe disease. These findings may help to elucidate the role of liver function tests on COVID-19 prognosis and provide a scope for improvement in the clinical treatment of patients during this current pandemic [41].

In conclusion, compared to nonsevere COVID-19, severe or critical COVID-19 is associated with increased markers of the innate immune response such as neutrophil count, NLR, IL-6, CRP, and serum ferritin; decreased markers of the adaptive immune response such as lymphocyte, CD4 and CD8 counts; and increased markers of tissue damage and major organ failure including D-dimer LDH, Troponin I, CK-MB, AST, ALT, urea, and creatinine. Based on the results of our meta-analysis, especially promising markers are NLR, IL-6, serum ferritin, lymphocyte and CD4 counts, D-dimer, and troponin I. The clinical value of these markers should be explored further to assess the risk of severe or critical disease and to monitor the clinical course of COVID-19 [42].

These findings collectively indicate that all liver enzymes and coagulation probes of patients affected with SARS-CoV-2 infection are within the normal range. Future studies would benefit from the inclusion of a control group and report the number of patients with changes in levels of liver function abnormalities [43].

Our study confirms the importance of measuring liver enzyme concentrations at admission, then monitoring the concentrations in hospitalized patients with COVID-19. Seriously ill patients have significantly elevated AST and D-dimer concentrations, and these changes have prognostic implications in the SARS-CoV-2 disease course [44].

In summary, in this cohort of 3,381 patients, ALI was more common among the 2,273 patients with confirmed SARS-CoV-2 than among those with a similar presentation who tested negative. However, SLI with ALT peak >5 times the ULN occurred in only 6.4% of patients. These liver enzyme elevations were rarely associated with cholestasis but did correlate with other markers of and-organ injury as well as cytokines and markers of inflammation. Finally, SLI was associated with the most severe clinical outcomes, including death, and may be a useful prognostic marker for hospitalized patients [45].

## CONCLUSION

Urea, creatinine, total bilirubin, SGPT, RBC, HGB, MCV, MCH, MPV, PDW, and DLC showed a significant change when compared with control in untreated COVID-19 patients. Urea and creatinine showed high mortality, an increase in ALT level, and a decrease in total bilirubin level, which indicates the changes observed in LFT by the viral attack of both populations. NE%, LY%, MO%, EO%, and RBCs show low values, and MCV and MCH show decreased values indicating severe COVID-19 abnormalities like pneumonia, aortic thrombosis, renal infarction/renal impairment, kidney impairment, acute tubular injury, kidney dysfunction, Liver injury due to enzymes and markers. Curcumin decreases the severity of COVID-19 by increasing MCV and MCH along with all parameters and cells. Thus, Curcumin can be used as a remedy/treatment to cure COVID-19.

## Figures and Tables

**Fig. (1a):**
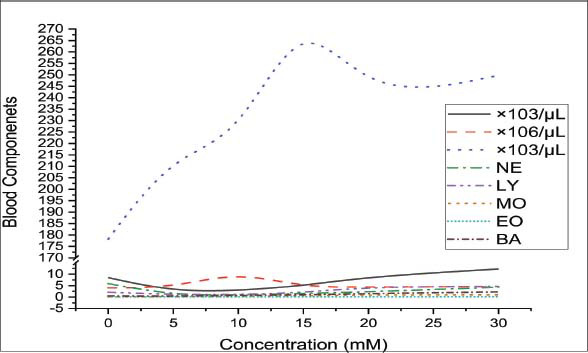
No. of cells (10^3^/µL) *vs*. curcumin concentration (mM).

**Fig. (1b):**
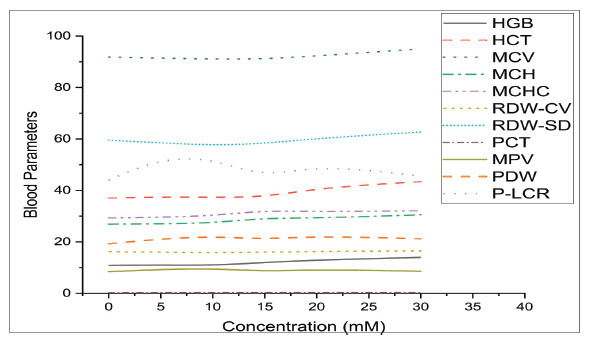
Blood parameters *vs*. curcumin concentration (mM).

**Fig. (2):**
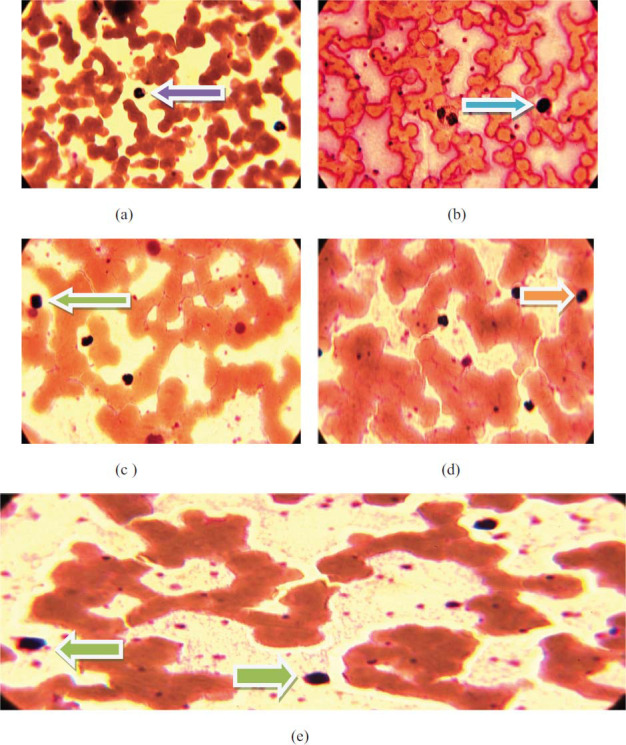
2-D images showing microscopic morphology of cells at 100X of each sample prepared with 3 mL blood of COVID-19 in EDTA tubes for (**a**) 0 mM Curcumin concentration, (**b**) 5 mM (0.005 mg) Curcumin concentration, (**c**) 10 mM (0.01 mg) Curcumin concentration, (**d**) 15 mM (0.015 mg) Curcumin concentration, (**e**) 20 mM (0.02 mg) Curcumin concentration.

**Table 1:** Comprehensive results of biochemical and CBC parameters.

**Parameters**	**COVID 19**	**(Control Group)**	***P* Value**
^b^Urea	36.72 ± 14.36	21 ± 7.88	0.000
^a^Creatinine	0.66 ± 0.26	0.84 ± 0.26	0.02
^b^Total Bilirubin	0.56 ± 0.26	0.85 ± 0.33	0.000
^b^SGPT (ALT)	58.84 ± 55.71	21.37 ± 11.08	0.000
^b^Alkaline phosphatase (ALP)	248.46 ± 75.42	210.91 ± 56.23	0.82
^b^Sodium	142.45 ± 1.92	139.26 ± 4.06	0.08
^b^Potassium	4.13 ± 0.19	4.35 ± 0.35	0.10
WBC×10^3^/µL	8.6 ± 4.4	7.7 ± 1.75	0.31
RBC× 10^6^/µL	4.78 ± 0.41	5.31 ± 0.41	0.001
HGBg/dl	13.2 ± 2.14	14.99 ± 1.06	0.001
HCT%	44.6 ± 3.81	46.46 ± 2.66	0.11
MCVfL	84.11 ± 40.94	87.99 ± 4.26	0.002
MCHpg	26.74 ± 12.96	28.54 ± 1.47	0.027
MCHCg/dl	27.92 ± 15.35	32.41 ± 0.65	0.12
PLT×10^3^/µL	217 ± 75.76	252.7 ± 63.83	0.05
MPV fL	11.05 ± 1.03	7.79 ± 0.67	0.00
PDW%	14.74 ± 2.59	19.86 ± 7.95	0.00
NE%	69.51 ± 14.38	53.26 ± 11.12	0.00
LY%	23.10 ± 12.98	38.68 ± 13.5	0.00
MO%	5.23 ± 3.98	0.72 ± 1.58	0.00
EO%	2.71 ± 1.53	0.56 ± 0.71	0.00

**Note:** ^a^Independent t-test, ^b^Mann-Witney U Wilcoxon test, ^*^significant result.

**Table 2:** The correlation table.

**Categories**	**Overall Results**						
**Parameters**	**Urea**	**Creatinine**	**Total Bilirubin**	**SGPT(ALT)**	**Alkaline Phosphatase (ALP)**	**Sodium**	**Potassium**
WBC10^3^/µL	-	-	-	-	-	-0.6 (0.04)	-
RBC10^6^/µL	-	-	-	-	-	-	-
HGBg/dl	-0.43 (0.04)	-	-	-	-	-	-
PDW%	-	-	-	-	-0.9 (0.03)	-	-
NE%	-	-	-	-	-	-	0.64 (0.03)
LY%	-	-	-	-	-	-	-0.5 (0.09)
MO%	-	-	-	-	-	-	-0.75 (0.007)
EO%	-	-	-	-	-	-	-0.83 (0.002)
**Pakistani Population**							
**Parameters**	**Urea**	**Creatinine**	**Total Bilirubin**	**SGPT`(ALT)**	**Alkaline Phosphatase(ALP)**	**Sodium**	**Potassium**
PLT10^3^/µL	-	-	-	-	-	0.85 (0.03)	-
NE%	-	-	-	0.82(0.007)	-	-	-
LY%	-	-	-	-0.83(0.006)	-	-	-
EO%	-	-	-	-	-	-0.92 (0.03)	-
**Chinese Population**							
**Parameters**	**Urea**	**Creatinine**	**Total Bilirubin**	**SGPT (ALT)**	**Alkaline Phosphatase (ALP)**	**Sodium**	**Potassium**
WBC10^3^/µL	-0.53(0.03)	-0.51(0.04)	-	-	-	0.96 (0.009)	0.97(0.006)
RBC10^6^/µL	-	0.86(0.03)	-	-	-	-0.95 (0.01)	-
HGBg/dl	0.73(0.001)	0.81(0.00)	0.72 (0.002)	0.64 (0.007)	-	-0.96 (0.007)	-0.94(0.02)
PLT10^3^/µL	-	-	-	-	-	0.89 (0.04)	-
NE%	-	-	-	-	-	0.92 (0.02)	-
LY%	-	-	-	-	-	-	-0.90(0.03)
MO%	-	-	-	-	0.51(0.04)	-	-
EO%	-	-	-	-	-0.62(0.01)	-	-

**Table 3:** Changes in blood parameters and cell count relative to curcumin concentration.

**Parameters**	**Normal Range**	**1^st^ Conc. (Without Curcumin) 0 mM**	**2^nd^ Conc. 5 mM**	**3^rd^ Conc. 10 mM**	**4^th^Conc. 15 mM**	**5^th^Conc. 20 mM**	**5^th^ (2^nd^ Time) After 30 Minutes**
WBC×10^3^/µL	4.00 – 11.0	8.5	2.41	2.75	4.98	8.94	12.25
RBC×10^6^/µL	4.20 – 6.30	4.03	4.09	11.21	4.10	4.43	4.57
HGB g/dl	12.0 – 18.0	10.84	11.04	10.84	12.00	12.94	13.94
HCT %	26.0 – 52.0	37.0	37.4	37.3	37.3	40.9	43.4
MCV fL	77.0 – 96.0	91.8	91.4	91.0	91.0	92.3	95.0
MCH pg	26.0 – 32.0	26.9	27.0	27.3	29.3	29.2	30.5
MCHC g/dl	32.0 – 36.0	29.3	29.5	30.1	32.2	31.6	32.1
RDW-CV %	11.6 – 14.0	16.2	16.0	15.8	16.0	16.3	16.5
RDW-SD fL	42.8 – 51.0	59.5	58.5	57.5	58.2	60.2	62.7
PLT×10^3^/µL	150 – 400	178.2	215.4	221.5	280.3	238.3	249.7
PCT %	0.16 – 0.33	0.15	0.20	0.21	0.24	0.22	0.21
MPV fL	7.00 – 11.0	8.4	9.3	9.7	8.5	9.3	8.6
PDW %	15.5 – 18.9	19.2	21.1	22.2	20.9	22.3	21.2
P-LCR %	20.0 – 58.0	44.0	52.6	52.6	44.7	50.1	45.6
NE %	1.10 – 7.00	5.87	0.75	0.87	1.53	2.23	4.30
LY %	0.70 – 5.10	2.10	1.03	0.96	1.89	4.42	4.69
MO %	0.00 – 0.90	0.13	0.18	0.27	0.58	0.92	0.96
EO %	0.00 – 0.90	0.00	0.07	0.00	0.07	0.07	0.10
BA %	0.00 – 0.20	0.45	0.38	0.65	0.91	1.48	2.20

## Data Availability

All the data and supportive information are provided within the article.

## LIST OF ABBREVIATIONS

CBC: Complete Blood Count
LFT: Liver Function Tests
MCH: Mean Corpuscular Hemoglobin
MCHC: Mean Corpuscular Hemoglobin Concentration
MCV: Mean Corpuscular Volume
MPV: Mean Platelet Volume
PCT: Platelet Crit
PDW: Platelet Distribution Width
PLT: Platelet Blood Count
RDW: Red Blood Cell Distribution Width
RPM: Renal Parameters
NETS: Neutrophil Extracellular Traps

## STANDARDS OF REPORTING

STROBE guidelines were followed.

## DECLARATION

Moreover, we declare that this is our original work and is not published previously.
